# Blood biomarkers, infrared thermography, and meat quality in Nellore bulls under tropical conditions

**DOI:** 10.1007/s11250-026-05009-6

**Published:** 2026-03-26

**Authors:** Guilherme Agostinis Ferreira, Amanda Gobeti Barro, Daniela Kaizer Terto, Karina Keller Marques da Costa Flaiban, Sérgio Bertelli Pflanzer, Ana Maria Bridi

**Affiliations:** 1 Departament of Animal Science, State University of Londrina (UEL), Rodovia Celso Garcia Cid PR 445 km 380, 86.057-970, Londrina, Brazil; 2 Graduate Program in Animal Science, Pontifícia Universidade Católica do Paraná (PUCPR), Curitiba, Brazil; 3 Department of Preventive Veterinary Medicine, State University of Londrina (UEL), Rodovia Celso Garcia Cid PR 445 km 380, 86.057-970, Londrina, Brazil; 4 Department of Food Technology, University of Campinas (UNICAMP), Rua Monteiro Lobato, 80, Campinas, São Paulo, 13083-862 Brazil

**Keywords:** Beef, Cortisol, DFD, Incidence, Myoglobin

## Abstract

This study investigated the use of stress-related blood biomarkers (cortisol, creatine kinase - CK, and lactate dehydrogenase - LDH) and infrared thermography (IRT) to predict meat quality in Nellore cattle under tropical conditions. These biomarkers reflect metabolic and stress pathways that influence muscle glycogen depletion before slaughter, contributing to elevated pHu and increased dark, firm, and dry (DFD) incidence. A total of 389 bulls from seven batches on the same farm were assessed at slaughter. Carcasses were classified by ultimate pH (pHu) as normal (pHu < 5.8), atypical DFD (5.8 ≤ pHu < 6.0), or typical DFD (pHu ≥ 6.0). The incidence of typical and atypical DFD meat was 20.8%. The typical DFD group showed significantly higher levels of cortisol, LDH, and CK. IRT images revealed that animals in the typical DFD group exhibited a higher minimum eye temperature (*P* < 0.003). In comparison, the atypical DFD group showed a higher maximum eye temperature compared to the normal group. Regression models demonstrated a strong predictive relationship (R² > 0.8) between cortisol, glucose, lactate, and pHu. We conclude that integrating blood biomarker analysis (specifically cortisol, glucose, and lactate) and IRT offers a practical tool for the early identification of DFD carcasses, which could enhance quality management in the beef industry.

## Introduction

The process of animal slaughter is complex and potentially stressful, involving various steps from farming, handling, transport, and lairage at the slaughterhouse (Gallo et al. 2022). These stressors may be acute or chronic in nature and can lead to physiological exhaustion, reduced energy reserves, and compromised meat quality, particularly increasing the risk of dark meat, or dark cutting (characterized by being dark, firm, and dry - DFD) (Warner et al. 2007; Ferguson and Warner 2008; Cappellozza and Marques 2021; Nelis et al. 2022) These challenges may intensify the animals’ physical effort and vigilance, leading to issues such as physical exhaustion and dark cutting (Ponnampalam et al. 2017).

Brazilian beef production is one of the largest in the international market, and meat is exported to several countries worldwide (ABIEC, 2024). The competitiveness of Brazilian beef in international markets depends not only on price but also on compliance with stringent standards for quality, traceability, and certification (Conter 2024). Among the determinants of beef quality, the final pH (pHu) is particularly important due to its influence on *post-mortem* biochemical processes; affecting protein functionality, water-holding capacity, color stability, microbial growth, and shelf life (Ponnampalam et al. 2017).

Despite its central role, countries differ substantially in the pHu thresholds used to classify DFD beef. For instance, in Europe and Australia, carcasses are graded as dark cutters when pHu exceeds 5.7 (Warner et al. 2014), 5.8 in Brazil, Canada, and Chile (Gallo 2004; Holdstock et al. 2014), 5.9 in the United States (McKenna et al. 2002), and up to 6.2 in Asian countries like China (Zhang et al. 2021). These variations are not based on consumer-derived visual acceptability, highlighting the need for science-based thresholds linking pHu, color measurements, and consumer preferenc. (Holman et al. 2017; Ponnampalam et al. 2017).

The Nellore breed of the *Bos taurus indicus* subfamily is the most commonly used among Brazilian beef cattle producers (Mueller et al. 2019), and exhibits characteristics that differ substantially from the European and crossbred cattle commonly found in North America, Europe, and Australia (Polkinghorne et al. 2008). Nellore cattle are known for greater behavioral reactivity, higher heat tolerance, lower muscle glycogen reserves, and a higher proportion of oxidative muscle fibers (Hooper et al. 2018; Aldrighi et al. 2019), which collectively increase their susceptibility to high pHu values, color instability, and DFD occurrence (Wheeler et al. 2010; Mahmood et al. 2017; Warner et al. 2022).

The DFD beef attributes are commonly associated with the final color of meat cuts, which exhibit high pH values and low lightness, redness, and yellowness levels (McKeith et al. 2016). The precise estimation of DFD meat incidence in Brazil or worldwide remains limited, varying from 1.3% to 13.9%, depending on the country (Ponnampalam et al. 2017), and can reach 25% (Hughes et al. 2017). In Brazil, the few available studies report regional incidence rates between 4% and 8% (Rosa et al. 2016; Barón et al. 2021).

However, the true incidence is likely higher because of substantial variability in cattle genotype, management and transport conditions, dietary systems, and slaughterhouse practices across production regions. This notable variation is especially relevant in the context of global meat trade, which reached 78.7 million tonnes (carcass weight equivalent) in 2024, largely driven by production and exports from major players such as Brazil, Australia, the European Union, China, and India (FAO 2025).

Pre-slaughter stress activates neuroendocrine pathways and alters circulating concentrations of glucose, lactate, cortisol, creatine kinase (CK), and lactate dehydrogenase (LDH), which reflect an animal’s physiological state and have been associated with dark-cutting predisposition (Egan et al. 2001; Muchenje et al. 2009; Probst et al. 2014; Nelis et al. 2022). This stress affects meat quality by depleting glycogen reserves, hindering lactic acid production post-mortem, and causing undesirable pH changes (Bourguet et al., 2015; Matarneh et al., 2023). Avoiding stress during this period is vital for beef quality and animal welfare (Lu et al. 2018).

In parallel, infrared thermography (IRT) has emerged as a non-invasive tool to assess the physiological stress response in animals (Ouyang et al. 2021). Since stress can trigger thermoregulatory changes, IRT allows for the remote measurement of skin surface temperature variations, particularly in regions like the eye, which is highly vascularized and located close to the brain (Ghezzi et al. 2024). Therefore, IRT offers a practical means to evaluate an animal’s stress state immediately before slaughter, providing a potential indicator of its physiological condition and its subsequent impact on meat quality.

To achieve a comprehensive understanding of meat production, it is crucial to investigate the behavior of animals, their production systems, and their impact on meat quality. Numerous studies have explored various factors influencing the complex parameter of meat pHu, including energy metabolism, oxidative processes, genomics, and proteomics ; (Gagaoua et al. 2018; Apaoblaza et al. 2020; Ijaz et al. 2020).

Despite the importance of Brazilian beef production, most studies addressing DFD incidence and pHu variability have focused on crossbred animals (Rosa et al. 2016) or *post-mortem* biochemical changes during ageing (Barón et al. 2021). Neither of these studies evaluated the predictive potential of pre-slaughter blood biomarkers or IRT, particularly in purebred Nellore bulls under tropical conditions. Therefore, the present study aimed to (i) quantify the incidence of beef with intermediate and high ultimate pH (pHu), and (ii) characterize the physicochemical differences among different pHu classes. In addition, we sought to (iii) evaluate the relationships between pHu, stress-related blood biomarkers (cortisol, glucose, lactate, CK, and LDH), and IRT measurements collected immediately before slaughter. Our central hypothesis was that animals exhibiting intermediate and high pHu values would show distinct physiological stress responses, reflected in both circulating biomarkers and IRT surface temperature patterns. We further hypothesized that the combination of these physiological and thermographic indicators could contribute to the early identification of carcasses at risk of producing DFD meat.

## Materials and methods

This study did not require approval from the institutional animal ethics committee, as all procedures were conducted in a commercial slaughterhouse under federal inspection, where blood sampling and carcass assessments were performed during routine industrial processing. The animals were processed according to the slaughterhouse’s standard operating procedures and protocols.

### Animals, slaughter, and experimental design

This study was conducted in March 2022 at a commercial slaughterhouse under federal inspection. The specific coordinates of the area are latitude 22° 14’ 6’” South and longitude 53° 19’ 54’” West, with an elevation of 401 m. The average temperature in the city is 23.9 °C, and the average annual rainfall and relative humidity are 1455 mm and 73%, respectively. During the three-day carcass collection period, the ambient temperature (26.1 ± 0.3 °C) and relative humidity (77.4 ± 0.5%) in the collection room were monitored using a data logger (HOBO^®^ U12-012).

A total of 389 Nellore bulls (24–38 months and an average cold carcass weight of 302 ± 12.4 kg) from seven slaughter batches were initially monitored. All animals originated from the same farm, and feeding systems and were subjected to the same preslaughter handling protocol, ensuring uniform management conditions across batches. The full dataset was required to quantify the overall incidence of DFD and to ensure the presence of variability in ultimate pH (pHu) values within and across batches.

**Fig. 1:**
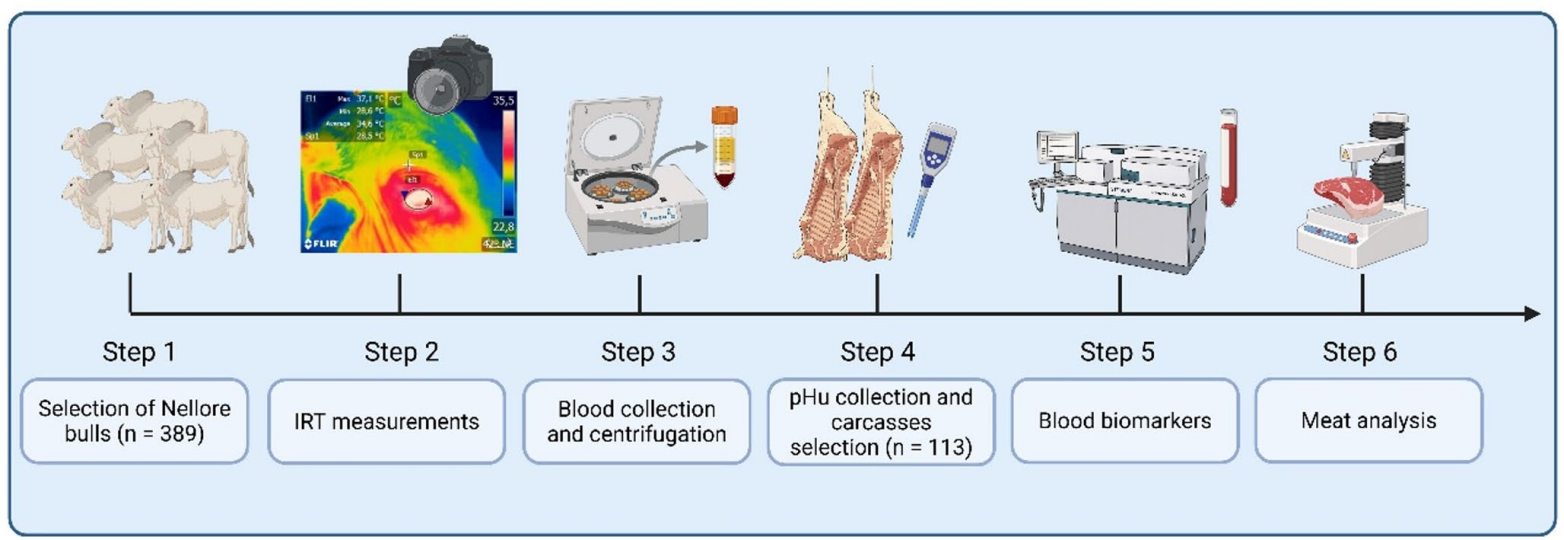
Schematic representation of the experimental workflow comprising six sequential stages: (1) Selection and evaluation of 389 Nellore bulls (*Bos taurus indicus*) from commercial finishing batches at abattoir; (2) Acquisition of infrared thermography (IRT) images at the stunning box for ocular temperature assessment; (3) Collection and centrifugation of blood samples immediately after exsanguination during the slaughter process; (4) Determination of ultimate pH (pHu) 48 h *postmortem* in the *Longissimus thoracis* muscle after carcass chilling, and selection of 113 carcasses classified into three pHu categories (normal, atypical, and typical dark, firm and dry – DFD beef); (5) Laboratory analyses of blood biomarkers (glucose, lactate, cortisol, creatine kinase, and lactate dehydrogenase); and (6) Meat quality parameters analysis (color, myoglobin, shear force, sarcomere length, and water loss)

Two of the seven monitored batches presented animals distributed across all three pHu categories evaluated in this study (normal, intermediate, and high pHu). From these two batches, a total of 113 animals were selected for the detailed physicochemical, biomarker, and IRT analyses to guarantee the presence of all pHu classes within each slaughter group (Fig. 1). The remaining batches, although monitored, did not contain animals spanning all pHu categories and were therefore excluded from the comparative statistical analysis. Nonetheless, they were included in the calculation of DFD incidence at the batch level.

The animals were slaughtered in accordance with animal welfare standards applied to establishments under federal inspection (MAPA, 2021). After 48 h of carcass chilling (4 °C), carcasses were evaluated based on their pHu values. The pHu was measured at the *Longissimus thoracis* muscle at the 12th rib using the Hanna HI98163^®^ professional pH meter (Hanna Instruments Inc, Barueri, Brazil). The pH meter was calibrated at chiller temperature beforehand using standard commercial solutions at pH 4 and 7.

The carcasses were classified into three pHu classes pre-established as experimental treatments, based on thresholds commonly used to characterize normal and dark-cutting beef. The first group, denoted as the “normal” group, consisted of carcasses with pH values of < 5.8. The second group, named the “atypical DFD” group, consisted of carcasses exhibiting pH values ranging 5.8–5.99. Finally, the third group, named the “typical DFD” group, encompassed carcasses with pH values of ≥ 6.0 (Table 1). The left carcasses were sectioned at the 12th rib to remove approximately 15 cm of the sample (*m. Longissimus thoracis*). Each portion was deboned and divided into steaks of varying sizes for subsequent analyses. The steaks were placed in polyamide/polyethylene bags, vacuum-packed, and frozen at -20 °C for transportation to the meat laboratory at the State University of Londrina and preserved until further analysis.

**Table 1:** Descriptive statistics (mean ± standard deviation, minimum, and maximum) of ultimate pH (pHu) measured in carcasses from Nellore bulls evaluated at a commercial slaughterhouse in Brazil

	*n*	pHu	Minimum	Maximum
Normal	37	5.68 ± 0.061	5.48	5.79
Atypical DFD	48	5.86 ± 0.058	5.80	5.98
Typical DFD	28	6.20 ± 0.189	6.00	6.94

Normal pHu (< 5.8). Atypical DFD (5.8 > pHu < 6.0). Typical DFD (pHu ≥ 6.0)

### Blood collection and measurements

Blood samples were collected during the bleeding and stored in 10 mL tubes (BD Vacutainers^®^, Curitiba, Paraná, Brazil) containing sodium fluoride for the evaluation of lactate and cortisol, and clot activator to assess levels of creatine kinase (CK) and lactate dehydrogenase (LDH). The determination of glucose, CK, LDH, and lactate levels was conducted with the Dimension^®^ Xpand Plus device (Siemens Healthcare Diagnostics Inc., USA) in duplicate using the following commercial kits: Gluc Ver Flex^®^ for glucose, CK-NAC IFCC^®^ for CK, LDI Flex^®^ for LDH, and Dimension LA^®^ for lactate, respectively. Cortisol concentrations in plasma samples were measured using a commercial cortisol ELISA kit (Jiaxing Korain Biotech Co., Zhejiang, China). The absorbance was determined using an ELISA reader (iMark™ Microplate Absorbance Spectrophotometer, Bio-Rad Laboratories Inc.) at 450 nm.

### Collection of infrared thermography (IRT) images

The eye temperature in live animals was non-invasively measured inside a stunning box at the slaughterhouse, adhering to standard industrial procedures. IRT images were obtained using a Flir T440 portable infrared camera (FLIR Systems Inc., Wilsonville, OR, USA) equipped with a 640 × 480-pixel detector and a 15° × 11° lens (40 mm) in the right eye and captured at a 90° angle from a distance of 60 cm. The camera had a thermal sensitivity of < 40 mK (< 0.04 °C at 30 °C ambient temperature) and a temperature range of -20–350 °C. The camera was set to automatic focus adjustment mode by a trained technician, and an emissivity value of 0.98— recommended for mammalian studies — was used (Bernard et al. 2013). The FLIR TOOLS^®^ software (Teledyne FLIR LLC, Wilsonville, Oregon, USA) was used to analyze the IRT images and obtain the maximum temperature (°C) within specific eye areas, including the posterior medial palpebral border, lower lid, and lacrimal caruncle.

### Meat color

Color analysis was performed after portioning the steaks and before freezing the samples. Beef samples were exposed to oxygen for 30 min at 4 ℃ (blooming time). The color was measured using the CIELab system of the CR-10^®^ colorimeter (Konica Minolta, Inc., São Paulo, Brazil), illuminant D65, observation angle of 10°, and opening size of 8.0 mm, performing three readings on the surface of the LT muscle sample. An average of three measurements was generated for each variable (L*, a*, and b*), where L* = lightness, a* = redness, and b* = yellowness. The colorimetric chromatic index (C*), which determines the color saturation, was calculated by the formula [(a*)^2^ + (b*)^2^] 0.5, and the hue angle (Hº) by the formula [tang^–1^ (b */a*) × [360°/ (2 × 3.14)] (MINOLTA, 1993).

### Thawing, cooking loss, and shear force

The samples were weighed before freezing and after thawing to determine the fluid loss. Next, a thermocouple was inserted in the geometric center of the samples, coupled to a digital thermometer ThermPro 4^®^ Probes Tp17h to monitor the internal temperature of the samples. The steaks were roasted in a preheated oven ate 170 °C (Paniz Indústria de Equipamentos para Comida Ltd., Caxias do Sul, RS, Brazil) equipped with a thermostat to avoid temperature fluctuations. When the internal temperature of the steak reached 40 ℃, the sample was turned over and remained in the oven until it reached an internal temperature of 71 ℃, based on the methodology described by Wheeler et al. (1998). Then, the samples were refrigerated at 4 ℃ for 24 h and weighed. Cooking loss was defined as the difference in weight before and after cooking.

After cooling, eight cylinders measuring 1.27 cm in diameter were removed in the direction of the muscle fiber using a punch attached to an industrial drill FSB 16 Pratika^®^ (Schultz Compressores S.A., Joinville, Brazil). The cylinders were sheared using a Warner-Bratzler shear blade coupled to a Brookfield CT-3 Texture Analyzer (Brookfield, São Paulo, Brazil) at a pre-load force of 2 N and a crosshead speed of 200 mm per min during shear.

### Sarcomere length

For each sample, a 2 × 2 × 1 cm piece was cut along the fiber direction. The piece was fixed for 4 h in a glutaraldehyde solution in 5% buffer (0.1 M Na_2_HP0_4_, pH 7.2). After a 4-hour incubation period, the samples were washed and kept with a 0.2 M sucrose buffer (0.1 M Na2HP04, pH 7.2), where they were preserved (4 °C) for no more than two days prior to measurements (Koolmees et al. 1986). Using fine-tipped tweezers, a small muscle bundle was extracted in alignment with the fiber direction. The isolated bundle was then positioned on a microscope slide with a drop of sucrose solution. The bundle was gently spread to distinguish a minimum of ten individual fibers. Subsequently, coverslips were applied to the slides, which were then positioned horizontally under a vertically aligned laser beam (Spectra Physics Inc., Model 117 A, CA, USA) to produce a sequence of diffraction bands. Sarcomere lengths were determined using the following formula:

$${\rm{Sarcomere}}\,{\rm{length}}\,{\rm{ \mu m = }}\left( {{\rm{0}}{\rm{.0006328 \times D \times }}\sqrt {} \left( {{\rm{T/D}}} \right){\rm{ + 1}}} \right){\rm{/T \times 1000}}$$

Where “D” is the distance (mm) between the blade and the measurement site, and “T” is the measured distance between two diffraction bands (mm).

### Myoglobin estimation

Myoglobin levels in the samples were estimated according to the methods described in the previous studies by Warriss et al. (1979) and Tang et al. (2004). The dilution factor was computed by dividing the volume of buffer (mL) by the mass (g) of the sample. The ratios of oxy-myoglobin (OMb), deoxy-myoglobin (DMb), and met-myoglobin (MMb) were calculated as described by Tang et al. (Tang et al. 2004), and the total myoglobin concentration was estimated using the following equation:

$$\eqalign{{\rm{Mb }}\left( {{\rm{mg}}\,{\rm{ g - 1}}} \right){\rm{ = }} & \left[ {\left( {{\rm{Absorption }}\,{\rm{at 525 -- Absorption}}\,{\rm{ at 730}}} \right){\rm{ / 2}}{\rm{.2303}}} \right]{\rm{ }} \cr & {\rm{ \times }}\left[ {{\rm{dilution}}\,{\rm{factor}}} \right] \cr}$$

### Statistical analysis

Statistical analysis was performed using the Jamovi^®^ program (The Jamovi Project, version 2.2). All variables obtained (biochemical markers, IRT temperatures, and meat quality traits) were continuous. Each carcass served as an experimental unit, and the pHu groups were treated as fixed effects, and the collection day and batch as random effects. Prior to analysis, data were screened for outliers using boxplot inspection. Outliers were removed only when associated with clear analytical or recording errors. The assumptions of normality and homogeneity of variances were tested using the Shapiro–Wilk and Levene’s tests, respectively. The pH data were subjected to descriptive statistical analysis in order to summarize the central distribution and variability of the sample. For the analysis of biochemical parameters and meat quality, one-factor analysis of variance (ANOVA) was performed, with statistical significance set at *P* < 0.05. *Post hoc* analysis was conducted using Tukey’s test to determine the specific pairwise differences between the groups. Unless otherwise stated, descriptive data are presented as mean ± standard deviation (SD), whereas error bars in figures represent the standard error of the mean (SE).

To understand the relationship between serum and plasma biomarkers and beef pHu, both principal component analysis (PCA) and linear regression analyses were conducted, considering the observed pHu values. To minimize the risk of overfitting due to high R² values, the models were validated using cross-validation procedures. For the regression models, ultimate pH (pHu) was used as the fixed effect, and the collection day and batch were considered as random terms for all covariates (lactate, glucose, CK, LDH, and cortisol), which were analyzed individually and in different combinations. The general structure of the regression model was:

$${{\rm{Y}}_{{\rm{ij}}}}{\rm{ = }}{{\rm{\beta }}_{\rm{0}}}{\rm{ + }}{{\rm{\beta }}_{\rm{1}}}{{\rm{X}}_{\rm{1}}}{\rm{ + }}{{\rm{u}}_{\rm{j}}}{\rm{ + }}{{\rm{\varepsilon }}_{{\rm{ij}}}},$$

where Y_i_ⱼ = ultimate pH of animal i in batch/day j; X_i_ = biomarker(s) or IRT parameter(s) used as predictors; β₀ = intercept; β₁ = fixed regression coefficient(s); uⱼ = random effect for batch and collection day; ε_i_ⱼ = residual error.

## Results and discussion

### Incidence and group pHu

For sample collection, 389 Nellore cattle carcasses were evaluated, and the incidence of carcasses presenting with a DFD anomaly was calculated based on this total. Considering only carcasses with a pHu ≥ 6, the incidence was 7.2%. However, when atypical DFD carcasses were included in the assessment, the percentage increased to 20.8%. These values represent a relatively high incidence, particularly considering that most commercial systems aim for rates below 5%.

Although high, these results fall within the broad range reported worldwide. Lower incidences have been reported in the United States, 3.2% (Moore et al. 2012), and in Brazil for crossbred heifers and imunocastrated Nellore x South African Simmental, Rosa et al.(2016) reported 4.53%. Intermediate values have been observed in Spain, where Mach et al.(2008) evaluated different breeds during the four seasons of the year, and reported 13.9%, reaching 17.1% when considering carcasses with a pHu above 6. Higher and more variable incidences are reported in Australia, where Hughes et al. (2017), in a study conducted on Australian plants, reported an overall DFD incidence of 12%, with variation ranging from 0.6% to 40.1% among plants, particularly those with a known history of higher DFD occurrence. Similar values, ranging 17–40%, were reported by Gallo(Gallo 2004) in a study onducted in Chile.

This observational study was conducted under commercial conditions using a single breed (Nellore) and slaughter facility, which limits causal inference and the extrapolation of results to other production systems. Nevertheless, this approach provides consistent and industry-relevant insights into pHu variation and DFD risk in Nellore cattle under tropical conditions.

### Blood variables

There was a significant difference between the groups with different pHu values and all blood parameters evaluated (Table 2). Blood cortisol levels were higher in the typical DFD group than in the normal and atypical DFD groups, but no differences were observed in these last two groups.

**Table 2:** Infrared thermography (IRT) measurements and blood stress-related biomarkers (mean ± standard error) in Nellore bulls classified according to three ultimate pH (pHu) ranges at slaughter

Parameter	Normal pHu < 5.8	Atypical DFD 5.8 > pHu < 6.0	Typical DFD pHu ≥ 6.0	*P* - value
IRTmin (ºC)	28.12 ± 0.18^a^	28.25 ± 0.16^a^	29.17 ± 0.25^b^	0.003
IRTmax (ºC)	35.30 ± 0.12^a^	36.01 ± 0.16^b^	35.87 ± 0.16^ab^	0.002
Cortisol (nmol/L)	20.48 ± 1.04^a^	21.13 ± 1.25^a^	29.33 ± 2.63^b^	0.019
Glucose (mmol/L)	117.12 ± 3.71^b^	103.90 ± 2.21^a^	115.52 ± 4.73^ab^	0.005
Lactate (mmol/L)	5.88 ± 0.30^a^	6.91 ± 0.26^ab^	7.79 ± 0.72^b^	0.01
CK (U/L)	612.2 ± 31.5^a^	875.8 ± 66.5^b^	910.0 ± 70.3^b^	0.001
LDH (U/L)	1366.09 ± 15.25^a^	1464.21 ± 23.48^b^	1479.94 ± 34.76^b^	< 0.001

Different letters on the same line represent a significant difference (*P* < 0.05). CK = creatine kinase; LDH = lactate dehydrogenase. CK: creatine kinase. LDH: lactate dehydrogenase. IRTmin: Minimum Temperature of the region of interest. IRTmax: Maximum temperature of the region of interest

The cortisol concentration in blood plasma is used to identify (acute) stress conditions in animals because it is released by the hypothalamic-adrenocortical axis, which is the main neuroendocrine system that responds to stressful situations, such as pre-slaughter stress (Foury et al. 2011; Russell et al. 2012). Activation of this system leads to the release of cortisol and a consequent increase in blood concentration. Pre-slaughter stress has been indicated as the primary cause of high pHu in cattle by causing muscle glycogen depletion, also resulting in a reduced amount of muscle lactate (Lu et al. 2018), and a rise of blood lactate due to the greatest mobilization of glycogen in the period immediately prior to slaughter (Coombes et al. 2014), fact also observed in this study.

Studies have shown that transport, lairage, and weaning interfere with cortisol concentration due to the mobilization of the animals’ energy reserves (O’Loughlin et al. 2014; Chulayo et al. 2016; Lu et al. 2018; García-Torres et al. 2021), leading us to believe that they may explain the results obtained in this study as well, since breed and sexual class factors can be discarded from this population because they are all male Nellore cattle.

Blood glucose levels were highest in the normal group, lowest in the atypical DFD group, and intermediate in the typical DFD group, which did not differ significantly from either extreme (normal or atypical). An opposite result was observed for blood lactate levels, with a lower level in the normal group than in the typical DFD group, with no difference between the atypical DFD group and the other two groups.

Previous studies have shown that animals exposed to pre-slaughter stress tend to exhibit higher blood glucose and lactate levels (Werner et al. 2013). Lactate concentration rapidly increases in response to physical and behavioral reactions to stress. Activation of the sympatho-adrenal system by pre-slaughter stress prompts the release of catecholamines from the adrenal medulla. Consequently, glycogen in the liver undergoes rapid breakdown, leading to elevated levels of blood glucose and lactate (Warriss 2010). In contrast, prolonged or late pre-slaughter stress, such as extended transport, may decrease glucose availability while increasing cortisol concentrations (Chulayo et al. 2016). This leads us to believe that the results of this study may be related to the greater susceptibility of the typical DFD group to pre-slaughter stress.

Considering this context, the patterns observed in our study in the typical DFD group (Table 2) are consistent with greater susceptibility to both early and late pre-slaughter stressors. However, because the study is observational, it is not possible to determine the exact timing or nature of the stress events, nor to establish a direct cause-and-effect relationship between stress and DFD. Instead, the results reinforce the biological plausibility that animals in the typical DFD group experienced more intense or cumulative stress before slaughter, contributing to their higher pHu values.

The amount of CK was higher in the typical and atypical DFD groups than in the normal group. The same was true for the amount of LDH. CK is an enzyme that occurs exclusively in muscle cells that catalyzes the phosphorylation of creatine to phosphocreatine and ADP to ATP (Brancaccio et al. 2007; Lu et al. 2018). Blood CK and LDH levels indicate tissue injury, increasing cellular permeability that can occur during various operations in pre-slaughter handling, such as physical stress caused by transport, injuries, loading, and unloading problems, or poor handling of animals in the slaughterhouse (Baird et al. 2012; Ekiz et al. 2012; Chulayo and Muchenje 2017).

Injuries incurred during the pre-slaughter period rapidly enter the bloodstream and serve as indicative markers of stress associated with fatigue and muscle metabolism (Lu et al. 2018). In a previous study, CK activity increased after animal transport, suggesting that preslaughter stress factors may increase its activity in cattle (Werner et al. 2013). Just as increased CK activity can be related to pre-slaughter stress, extracellular LDH can also be detected, as it is an enzyme detected in the blood after vigorous exercise or muscle trauma (Lu et al. 2018). The results of this study are in line with those of previous studies that indicate an increase in the activities of these enzymes in stressful situations and are positively correlated with pHu (Lu et al. 2018; García-Torres et al. 2021).

### IRT measures

Temperature variables varied among the groups. Animals in the typical DFD group exhibited higher minimum temperatures than those in the other two groups. When analyzing the maximum ocular IRT, the atypical DFD group presented higher temperatures than the normal group and did not differ from the typical DFD group. No significant differences were observed between the typical DFD and normal groups.

Cuthbertson et al. (2020) studied the use of IRT on a farm video to predict meat quality and observed that animals with higher temperatures had meat with elevated pHu and a darker color, similar to what we found concerning IRTmin. The authors hypothesized that this was because stressed cattle use glycogen stores in their muscles to maintain homeostasis, which can cause an increase in brain temperature. This temperature rise can be identified through eye IRT, because the eyes are close to the brain (Tang et al. 2008). After slaughter, glycogen reserves are converted into pyruvate, resulting in the production of lactic acid. Lactic acid produces hydrogen ions, which decrease meat pH. Consequently, inadequate production of lactic acid due to depleted glycogen stores at slaughter can lead to high meat pH, resulting in DFD (Devine et al. 2006; Gregory 2008).

Limited research has been conducted on the potential application of IRT to predict carcass quality and meat characteristics, particularly in live animals. A study conducted in Spain (Horcada et al. 2019), revealed a correlation between IRT measurements (maximum temperature) and pHu of light carcasses. Another Canadian study (Schaefer et al. 2018), utilized real-time IRT technology and found that normal animals had an average ocular temperature of 33.6 °C, whereas animals with DFD carcasses displayed lower and higher temperatures (30.4 ± 1.85 and 35.8 ± 1.28 °C, respectively).

However, in our study, animals with typical DFD exhibited higher IRTmin, while both atypical and typical DFD carcasses showed identical IRTmax. Only atypical DFD carcasses differed from the normal group. Notably, the exact timing of image acquisition in the study by Schaefer et al. (Schaefer et al. 2018) remains unclear. Our findings indicate that normal animals had higher ocular temperatures than those reported by Schaefer et al. (2018).

Because the study was conducted under tropical conditions, elevated ambient temperature and humidity likely contributed to the higher absolute IRT values observed across all animals. Importantly, however, all animals (regardless of pHu group) were held in the same lairage pens and passed through the stunning corridor under identical environmental conditions. Thus, although ambient temperature may influence baseline surface temperature, it does not account for the differences detected between pHu groups. The absence of precise ambient temperature records nevertheless represents a limitation and should be considered when interpreting the thermographic results.

### Meat quality

The results obtained for the color parameters support the hypothesis that pHu affects not only lightness but also other variables (Table 3). The variables L*, a*, b*, C*, and H* differed according to pHu groups. The L* and water loss means supported the existence of differences between steaks classified as atypical and typical DFD, except for cooking loss, which did not show differences between the groups (*P* = 0.3). The atypical DFD steaks had similar color characteristics as the typical DFD steaks, showing lower values of redness and yellowness than the normal steaks. They differed only in L*, indicating that the atypical DFD group was intermediate between the other two groups.

**Table 3:** Meat traits (mean ± standard error) of Nellore bull carcasses grouped according to ultimate pH (pHu) categories

Parameters	Normal pHu < 5.8	Atypical DFD 5.8 > pHu < 6.0	Typical DFD pHu ≥ 6.0	*P* - value
L*	36.3^c^ ± 0.25	35.4^b^ ± 0.22	34.2^a^ ± 0.32	< 0.001
a*	11.9^b^ ± 0.32	9.7^a^ ± 0.31	8.6^a^ ± 0.29	< 0.001
b*	12.9^b^ ± 0.15	12.1^a^ ± 0.17	11.6^a^ ± 0.16	< 0.001
Hue angle	47.8^a^ ± 0.54	51.8^b^ ± 0.53	53.7^b^ ± 0.62	< 0.001
Chroma	17.8^b^ ± 0.32	15.5^a^ ± 0.32	14.6^a^ ± 0.32	< 0.001
Total Mb (mg g^− 1^)	4.67 ^c^ ± 0.103	4.08 ^b^ ± 0.096	3.70 ^a^ ± 0.128	< 0.001
OxyMb (%)	60.5 ^b^ ± 0.012	56.3 ^a^ ± 0.010	54.6 ^a^ ± 0.014	0.006
DeoMb (%)	9.8 ^a^ ± 0.003	10.6 ^ab^ ± 0.002	11.1 ^b^ ± 0.004	0.028
MetMb (%)	26.7 ^a^ ± 0.008	29.2 ^b^ ± 0.006	30.7 ^b^ ± 0.007	0.003
WBSF (N)	92.87^b^ ± 0.40	100.22^b^ ± 0.37	71.49^a^ ± 0.43	< 0.001
Sarcomere length (µm)	1.62 ^a^ ± 0.013	1.63 ^a^ ± 0.014	1.70 ^b^ ± 0.014	< 0.001
Thawing loss (%)	14.20^b^ ± 0.61	13.94^b^ ± 0.46	9.83^a^ ± 0.85	< 0.001
Cooking loss (%)	20.99 ± 0.47	19.95 ± 0.44	20.46 ± 0.66	0.289

Different letters on the same line represent a significant difference (*P* < 0.05). Normal pHu (< 5.8). Atypical DFD (5.8 > pHu < 6.0). Typical DFD (pHu ≥ 6.0). L* = lightness. a* = redness. b* = yellowness. DeoMb = deoxymyoglobin. OxyMb = deoxymyoglobin. MetMb = metmyoglobin. Mb = myoglobin

The color of meat is not just an aesthetic feature but can also provide information on its quality and freshness. The normal group showed a fresh red appearance, which can be attributed to metabolic processes occurring during the *postmortem* period of the muscle (Ramanathan et al. 2019).

Our findings are consistent with those of Li et al. (2018), who revealed that the majority of proteins related to color stability were glycolytic enzymes and that myoglobin phosphorylation was inversely related to meat color stability. As the level of myoglobin phosphorylation increased, the color stability of meat, based on the a^∗^ value, decreased, and the proportion of metmyoglobin (MetMb) increased. Furthermore, we found an inverse relationship between pHu and some parameters related to meat color, including a*, b*, chroma. As pHu increased, there was a decrease in the values of these parameters and a greater MetMb proportion.

There were significant differences in the total myoglobin concentrations among the three groups (*P* < 0.001). The normal group exhibited higher average myoglobin concentration than the atypical and typical DFD groups, which also showed distinct values. Additionally, the oxygenated Mb (OxyMb) proportion was higher in the normal group, whereas no difference was observed between the atypical and typical DFD groups. Conversely, the MetMb proportion was higher in the atypical and typical DFD groups than in the normal group, with no significant difference between the two groups. The terms of deoxymyoglobin Mb (DeoMb) proportion, the typical DFD group displayed a higher average than in the control group, whereas the atypical DFD group did not differ significantly from either group.

In the current study, steaks from the typical and atypical DFD group exhibited a lower proportion of OxyMb than normal group, which imparts a bright cherry-red color to the meat; a higher proportion of MetMb, which contributes to the brown color; and similar proportions of DeoMb to normal and typical DFD groups. These results may be related to the shorter sarcomere length (Table 3), which can result in the compression of the myofibrillar structure and a decrease in myoglobin oxygenation 74. The decrease in OxyMb and the higher proportion of MetMb in the typical DFD group may suggest a higher rate of oxygen consumption, a characteristic previously observed in dark-cut meats (Tang et al. 2005).

Contrary to the common misconception that DFD meat is tough due to the term “firm”, our evaluation results indicate that the meat from the typical DFD group was the most tender compared to all other groups examined. Interestingly, our findings suggest that the tenderness of steaks from the atypical DFD group was similar to that of steaks from the normal group. Furthermore, the greater sarcomere length observed in typical DFD steaks supports an inverse relationship between sarcomere length and shear force (Weaver et al. 2009). This finding aligns with that of a similar study conducted on Puerto Rican cattle, which also reported greater sarcomere length in the typical DFD group, without further discussion of possible reasons (Torres-Burgos et al. 2019). During the thawing process, a significant difference was observed, with steaks from the typical DFD group losing less water compared to the other two groups, which did not exhibit differences between them.

### The relationship between blood biomarkers and pHu

**Fig. 2:**
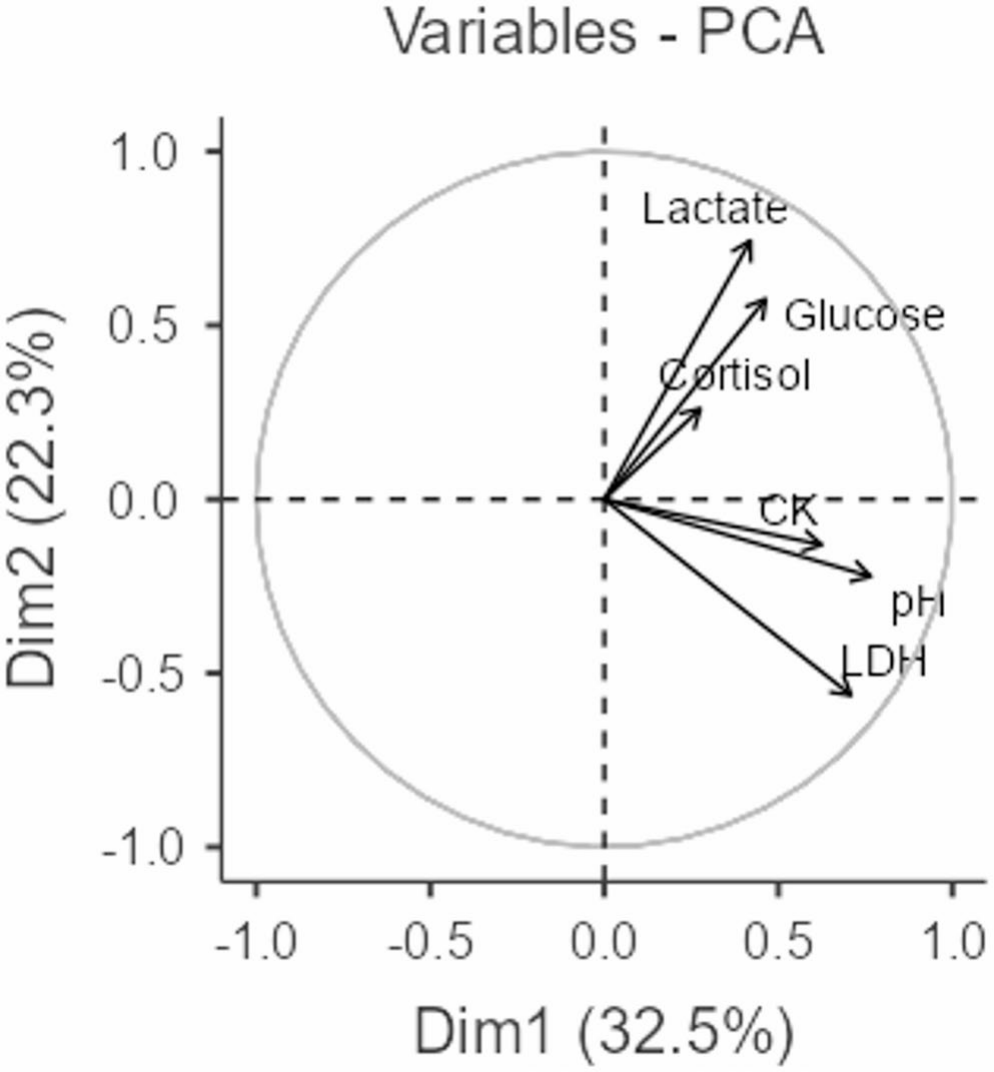
Principal component analysis (PCA) of stress-related blood biomarkers and infrared thermography (IRT) variables in Nellore bulls

In the present study, principal component analysis (PCA) revealed three main components (Fig. 2). The first component was characterized by the strong contribution of LDH variables, with a weight of 0.92, CK with a weight of 0.57, and pHu with a weight of 0.75. The high positive loadings of these variables suggest an association between a higher final pH and elevated concentrations of LDH and CK.

The second component was characterized by the variables CK, glucose, and lactate with respective weights of 0.35, 0.94, and 0.57. This component revealed that elevated levels of glucose are associated with CK and lactate, indicating a potential relationship between glucose metabolism and meat characteristics. This association may be related to the use of glucose as an energy source in muscular metabolic processes.

The third component showed a strong contribution of pHu with a weight of 0.40, lactate with a weight of 0.79, and cortisol with a weight of 0.58. This association suggests a relationship between the final meat pH, lactate concentration, and cortisol, which may be related to metabolic and stress processes during slaughter.

It is important to highlight that the absence of significant cross-loadings between these components strengthens the interpretability and validity of the observed relationships. The variance values of Component 1 (32.5), Component 2 (22.3), and Component 3 (17.2) indicate that these components capture a substantial part of the total variation in the data.

The results of the linear regression analyses (Table 4) demonstrate that blood biomarkers such as Lactate, Glucose, CK, LDH, and cortisol have significant associations with the pHu of beef, as indicated by the significant R² values ranging from 0.71 to 0.87 (*P* < 0.001). The adjusted R² was also considered to assess the ability of the predictors to explain the variation in the beef pHu while accounting for the number of predictors in the model. The high adjusted R² values indicate that the regression models are robust, and the selected predictors contribute significantly to the variation in the final pH. Furthermore, the Root Mean Square Error (RMSE) provides a measure of the accuracy of the regression models. RMSE was used as a measure of predictive error and interpreted in relation to the scale of the response variable (pHu) and comparatively among the tested models. Lower RMSE values indicate reduced prediction error and greater precision of the estimates, but do not, by themselves, imply overall model goodness-of-fit.

**Table 4:** Linear regression models evaluating the predictive capacity of individual and combined blood biomarkers for ultimate pH (pHu) in Nellore bulls slaughtered under commercial conditions

Predictors	*R* ^2^	*R*^2^ adjusted	RMSE	*P* - value
Lactate	0.78	-	0.11	< 0.001
Glucose	0.76	-	0.12	< 0.001
CK	0.72	-	0.13	< 0.001
LDH	0.71	-	0.12	< 0.001
Cortisol	0.87	-	0.08	< 0.001
Lactate + Cortisol	0.87	0.85	0.07	< 0.001
Cortisol + Glucose	0.88	0.86	0.08	< 0.001
Cortisol + Glucose + Lactate	0.86	0.80	0.08	< 0.001
Lac + Glu + Ck + LDH + Cor	0.83	0.68	0.06	0.015

CK = creatine kinase. LDH = lactate dehydrogenase. Glu = glucose. Cor = cortisol. R² = coefficient of determination. R² adjusted = adjusted coefficient of determination. RMSE = Root Mean Square Error

Among the single predictors, cortisol explained the greatest proportion of pHu variability (R² = 0.87), indicating that it is the strongest individual indicator of physiological stress related to high pH development, which is physiologically consistent, as cortisol reflects activation of the hypothalamic–pituitary–adrenal (HPA) axis during pre-slaughter stress (Russell et al. 2012). Lactate, glucose, CK, and LDH also showed substantial explanatory power (R² = 0.71–0.78), reflecting metabolic responses related to glycogen mobilization and anaerobic activity processes directly linked to muscle glycogen depletion and the development of high pHu (Gavin et al. 2015).

When biomarkers were combined, predictive capacity increased further. The models, including cortisol + glucose (R² = 0.88; adj. R² = 0.86) and cortisol + lactate (R² = 0.87; adj. R² = 0.85), showed the best overall performance, reinforcing that the interaction between endocrine (cortisol-mediated) and metabolic (glucose and lactate) stress responses is central to the development of elevated pHu. This is expected because animals experiencing greater acute or cumulative stress typically show simultaneous activation of the HPA axis and increased reliance on anaerobic metabolism, leading to reduced post-mortem lactic acid production and consequently higher pHu. These models also exhibited low RMSE values (0.07–0.08), indicating strong predictive accuracy.

In contrast, the full model including all five biomarkers (“Lac + Glu + CK + LDH + Cortisol”) performed worse despite having more predictors (R² = 0.83; adj. R² = 0.68; *P* = 0.015). This suggests that adding all biomarkers simultaneously introduces redundancy and may dilute the contribution of the most informative predictors, reducing overall model efficiency. Therefore, models combining cortisol with either glucose or lactate appear to provide the most physiologically coherent and statistically robust predictions of pHu.

The association of blood biomarker parameters in cattle presents different behaviors according to the breed and study design (Lu et al. 2018; García-Torres et al. 2021), but in general, it can vary from weak to moderate, and in some cases, it can be strong. However, it must be analyzed carefully when making comparisons between studies.

There are indications of an association between the pHu and blood cortisol levels. De Freslon et al. (De Freslon et al. 2014) evaluated the association of behavioral tests for the incidence of dark cutting, and the results showed a moderate correlation (*r* = 0.34; *P* < 0.05) between cortisol levels on the farm and crash test scores, which indicate the behavior of an animal. The study also revealed a high correlation (*r* = 0.81–0.87; *P* < 0.01) between the behavior test and pHu, indicating that combining physiological measurements with behavior observations could be beneficial.

Lu et al. (2018) reported a direct correlation between plasma lactate levels during exsanguination and dark cutting, with a correlation coefficient (r) of 0.80. Additionally, a strong correlation was identified between cortisol (*r* = 0.96), lactate dehydrogenase (*r* = 0.84), and creatine kinase (*r* = 0.97) plasma levels and intermediate correlations with glucose (*r* = 0.62) plasma levels and final pH.

The correlation studies mentioned above demonstrate associations between variables but do not establish a direct cause-and-effect relationship. Associations between blood biomarkers, animal behavior, and pHu can vary in intensity, ranging from weak to moderate, and in some cases, they can be strong. However, it is crucial to remember that correlation does not imply causality. Our prediction models were developed based on these observed associations and provided significant results in explaining the variation in meat pHu. However, they represent a predictive tool and do not establish definitive cause-and-effect relationships. Other factors not considered in our analysis may influence the results.

From a practical standpoint, the results highlight the potential of combining IRT and blood biomarker measurements collected during exsanguination as tools for the early identification of carcasses prone to atypical or typical DFD. In Brazilian slaughterhouses, these approaches could be integrated into quality control protocols to guide carcass allocation across different export markets, where specific pHu thresholds are required. Compared to Rosa et al. (2016), who reported a lower DFD incidence (4.53%) in crossbred cattle without evaluating physiological markers, our study demonstrates a higher incidence in purebred Nellore bulls and expands the analysis by incorporating objective biomarkers.

## Conclusion

Based on the findings of this study, it can be concluded that meat with atypical and typical DFD values differ in terms of meat quality traits. The analysis revealed variations in blood biomarkers such as cortisol, CK, LDH, glucose, and lactate among the pHu groups. The atypical and typical DFD groups exhibited higher MetMb proportion, showing an increase of 4%, which may impact meat color and oxidative stability. The typical DFD group showed the highest tenderness. An association between blood biomarkers and beef pHu has been established, with cortisol and lactate identified as the most relevant biomarkers. the integration of blood biomarker analysis and infrared thermography offers a promising avenue for early carcass triage, improving product allocation to domestic and international markets. Future studies should expand these findings by incorporating behavioral indicators, genomic information, and large-scale validation in commercial abattoirs to consolidate predictive systems for beef quality under tropical conditions.

## Data Availability

The data supporting this study are available from the corresponding author upon request.
